# Natural versus commercial carbohydrate supplementation and endurance running performance

**DOI:** 10.1186/1550-2783-9-27

**Published:** 2012-06-15

**Authors:** Brandon W Too, Sarah Cicai, Kali R Hockett, Elizabeth Applegate, Brian A Davis, Gretchen A Casazza

**Affiliations:** 1 Sports Performance Laboratory, University of California, Davis, Medical Center Sports Medicine Program, Sacramento, CA, USA; 2 Physical Medicine and Rehabilitation, University of California, Davis, Medical Center, Sacramento, CA, USA; 3 Department of Nutrition, University of California, Davis, Davis, CA, USA; 4 UC Davis Sports Medicine Program, 2805 J St., Suite 300, Sacramento, CA 95816, USA

**Keywords:** Blood glucose, Time trial, Respiratory exchange ratio, Creatine kinase, Insulin, Free fatty acid

## Abstract

**Background:**

We examined the metabolic, performance and gastrointestinal (GI) effects of supplementation with a natural food product (raisins) compared to a commercial product (sport chews).

**Methods:**

Eleven male (29.3 ± 7.9 yrs; mean and SD) runners completed three randomized trials (raisins, chews and water only) separated by seven days. Each trial consisted of 80-min (75%VO_2_max) treadmill running followed by a 5-km time trial (TT). Heart rate (HR), respiratory exchange ratio (RER), blood lactate, serum free fatty acids (FFA), glycerol and insulin, plasma glucose and creatine kinase, GI symptoms and rating of perceived exertion (RPE) were recorded every 20-min. We employed a within-subject two-way analysis of variance (ANOVA) for repeated measures with a Fisher’s post hoc analysis to determine significant differences.

**Results:**

VO_2_, HR, lactate, glycerol and RPE did not differ due to treatment. Average plasma glucose was maintained at resting levels (5.3 ± 0.4 mmol·L^-1^) during the sub-maximal exercise bout (5.9 ± 0.6, 5.7 ± 0.6 and 5.5 ± 0.5 mmol·L^-1^ for chews, raisins and water respectively), and was significantly higher with chews than water only. RER and % of non-protein macronutrient oxidation derived from carbohydrate was highest with chews, followed by raisins and water was the lowest (74.4 ± 6.4, 70.0 ± 7.0 and 65.1 ± 8.7% for chews, raisins and water respectively) during the sub-maximal exercise period. Serum FFA was higher in the water treatment versus both raisins and chews at 80 min of sub-maximal exercise. Serum insulin was higher with the chews than both raisins and water (5.1 ± 2.0, 3.1 ± 0.8, 1.9 ± 0.6 uU·ml^-1^ for chews, raisins and water respectively). Plasma creatine kinase, corrected for baseline values, for the last 40 min of the sub-maximal exercise bout, was higher with raisins compared to other treatments. The TT was faster for both carbohydrate supplements (20.6 ± 2.6, 20.7 ± 2.5, 21.6 ± 2.7 min for raisin, chews and water respectively). GI disturbance was mild for all treatments.

**Conclusion:**

Raisins and chews promoted higher carbohydrate oxidation and improved running performance compared to water only. Running performance was similar between the raisins and chews, with no significant GI differences.

## Background

It has been well established that carbohydrate (CHO) consumption before and during exercise improves exercise performance in events lasting longer than one hour, by maintaining blood glucose, high CHO oxidation rates and possibly sparing endogenous glycogen stores [1,2]. What is less clear is the relationship between the CHO amount, type and form to maximize endurance performance.

Early studies utilized single CHO types such as glucose or glucose polymers [2,3], but more recently the ingestion of a glucose plus fructose mixture has been shown to be more effective [1,4-7]. Ingestion of a glucose plus fructose drink had higher exogenous CHO oxidation rates compared to glucose or fructose only drinks due to increased intestinal absorption rate from both the sodium-dependent glucose (SGLT1), fructose (GLUT5), and glucose and fructose (GLUT2) intestinal transporters [1,6,8]. Ingestion of a mixed CHO source allows for greater CHO absorption and utilization, which can be beneficial during prolonged exercise.

More recently, researchers have investigated whether other CHO forms (solids and semisolids) have the same benefits as a liquid. No significant metabolic or exercise performance differences have been found when consuming solid or semisolid CHO sources before-exercise [9-11]. Previously in our lab, the effects of a sport drink, sport gel, sport beans and water were studied in trained cyclists during 80-min of exercise at 75% VO_2_max, showing no significant metabolic or performance differences between the commercial sport products [12]. A series of studies performed by Pfeiffer and colleagues also confirmed that the exogenous CHO oxidation rates between CHO delivery via fluid, semi-solid or solid were similar during 180-min of cycling at 58% VO_2_max [5,13].

As individuals decide to take a more whole food approach, other nutritional factors (e.g. dietary fiber) can affect CHO supplementation choice. The low digestibility of fiber can elicit an osmotic and fermentative effect in the intestinal lumen, which can have unwanted side effects such as flatulence, belching, nausea, abdominal pain and diarrhea [14]. The prevalence of gastrointestinal (GI) discomfort may increase when ingesting low digestible CHO combined with exercise, resulting in a decrease in performance. A study examining the effects of raisins versus sports gels as pre-exercise feedings in cyclists showed no significant metabolic or performance differences during a 45-min sub-maximal cycling bout at 70%VO_2_max followed by a 15-min performance trial [10]. We know of no study to examine the effects of raisins versus commercial sports products in runners. GI complaints are more pronounced during running, which may be related to the greater mechanical jarring involved [15]. Reports have also noted that 83% of marathoners and 81% of endurance athletes experience some level of GI distress during training or competition [15]. Ingesting a higher fiber supplement in raisins during an endurance run may cause more GI discomfort than eating lower fiber sports products.

Therefore, the purpose of this study was to examine the metabolic and running performance effects and GI tolerance of a natural whole food product (raisins) compared to a commercial product (sport chews) and water only. It was hypothesized that the raisins and chews would elicit similar metabolic responses and both would improve running time trial performance over water only, yet because of the higher fiber content, raisins would elicit greater GI discomfort.

## Methods

### Subjects

Fourteen healthy competitive runners were recruited from the University of California at Davis (UC Davis) campus and local venues. Twelve subjects were needed based on a power analysis (http://hedwig.mgh.harvard.edu/sample_size/js/js_crossover_quant.html) (power = 0.8, significance = 0.05, mean difference (MD) = 0.58 min for performance time of supplement versus water in men only and SD of the MD = 0.64 min) [12]. Three subjects quit during the study before all trials were completed for reasons unrelated to the supplementation (aversion to needles, calf strain, knee pain). Therefore, only 11 of 14 subject’s data were included in the analysis (power = 0.8). Subjects were required to have ran a marathon in <4-hr or completed two half marathons in <2-hr within the past year and run >48 km·week^-1^. Medical clearance and an informed consent approved by the UC Davis Institutional Review Board were also required.

### Training and diet

Subjects recorded all training sessions for the week prior to the first sub-maximal exercise test and repeated that same exercise program for the remainder of the study. Subjects were advised to rest or have a light training day prior to all testing days. The subjects’ general diets were monitored by a 3-day diet record completed before the first meeting. 24-hour recalls were completed the day prior to the first sub-maximal exercise trial and repeated exactly for all subsequent trials (Food Processor SQL Version 9.2.0, ESHA Research, Salem, OR). A 240-kcal snack (68% CHO, 16% fat and 16% protein) (Clif Bar, Berkeley, CA) was provided to consume 10-hr before each of their testing times. After the provided evening snack, only water was consumed.

### Maximal exercise test

Subjects reported to the laboratory for their first visit which included a medical clearance examination and maximal exercise test. Height and body mass were measured and body composition was determined via 7 sites and a Harpenden caliper [16]. Exercise tests were performed on a treadmill (Stairmaster Clubtrack, Vancouver, WA) set at 1% incline. After a 5-min warm-up, a graded exercise test to exhaustion was completed to determine maximal oxygen consumption (VO_2_max). The initial speed was based on their most recent marathon pace and increased every 2-min by 0.8-km·h^-1^ until volitional fatigue. A metabolic cart (TrueOne 2400, ParvoMedics, Sandy, UT) was used for metabolic measurements. At the end of every 2-min stage, heart rate (HR) via a HR monitor (5410, Polar, Woodbury, NY) and rate of perceived exertion (RPE) using a 10-point scale [17] were measured. The treadmill speed eliciting 75%VO_2_max was used as the starting speed for the sub-maximal exercise trials.

### Sub-maximal exercise trials

All sub-maximal trials were done 7–14 days apart. Subjects reported to the lab at ~8:15 am in a fasted state, under normal environmental conditions: 21-23 °C, 757–761 mmHg and 35-46% relative humidity. Subjects first completed the pre-exercise questionnaires: whole body muscle soreness and fatigue (marking a line on a 100 mm visual analogue scale from no pain to extreme pain or not tired to utterly exhausted) and a gastrointestinal discomfort questionnaire (GIDQ) created by our lab. The GIDQ included 7 categories (abdominal pain, heartburn, regurgitation, bloating, nausea, belching and flatulence) rated as 0 (none), 1 (mild), 2 (moderate), 3 (quite a lot), 4 (severe), 5 (very severe) and 6 (unbearable). A 22 G catheter was then inserted into a forearm vein for blood sampling. After 10-min rest, a 9-ml blood sample was obtained. A randomized nutritional treatment was given and then subjects performed the same 5-min warm up on the treadmill for all trials. This was followed by voiding and getting a pre-exercise body weight.

During the first 80-min of the first trial, the treadmill speed was adjusted to maintain 75%VO_2_max and the same treadmill speed increments were used for all subsequent trials. Every 20-min during the 80-min exercise bout, GI symptoms were recorded and a 9-ml blood sample was taken while the subject stopped and straddled the treadmill for ~2-min while consuming their treatment. HR, oxygen consumption (VO_2_), respiratory exchange ratio (RER) and RPE were measured during the 5-min prior to stoppages. Stopwatch time was paused during stoppages so subjects ran the full 80-min. Immediately after the 80-min, the subjects completed a 5-km TT where they controlled the speed. Only the total distance covered was shown to the subjects. The time to complete the TT and average RPE, GIDQ, and HR were recorded. After a 5-min active recovery, a post-exercise body weight was recorded. Immediate, 2-hr and 5-hr post-exercise questionnaires identical to the pre-exercise questionnaires were completed.

### Supplement formulation

One of two CHO supplements (pre-exercise: 0.5 g CHO·kg BW^-1^ and every 20-min during exercise: 0.2 g CHO·kg BW^-1^) or water only was randomly assigned for each week. CHO supplements included: #1 - raisins, (31 g (~1/5 cup)): 100-kcal, 24 g CHO (glucose and fructose in 1:1 ratio), 1.6 g fiber, 0.8 g protein, 8 mg sodium, 238 mg potassium and #2 – Chews (Clif blocks) (3 pieces, 30 g): 100-kcal, 24 g CHO (brown rice syrup (45% maltose, 3% glucose, and 52% maltotriose) and cane juice (50% glucose and 50% fructose)), 70 mg sodium and 20 mg potassium. Fluid intake was kept constant at 7 ml·kg BW^-1^ pre-exercise and 2.5 ml·kg BW^-1^ every 20-min during exercise for all treatments.

### Blood analysis

Blood samples were collected in non-heparinized syringes. One drop (~20 μl) measured blood lactate (Lactate Pro, Arkray, Inc, Kyoto, Japan) and hematocrit was determined using microhematocrit tubes (Statspin, Norwood, MA). 9-ml of blood was aliquoted into two SST tubes and one lithium heparin tube and was centrifuged at 3000 rpm for 15-min. 100 μl from the lithium heparin tube was analyzed for plasma glucose, sodium, potassium, and creatine kinase (CK) levels in a Metlyte 8 reagent disc (Piccolo Xpress Chemistry Analyzer, Abaxis, Union City, CA). Serum from the SST tubes was used for free fatty acid (FFA) (Wako Chemicals, Richmond, VA) and glycerol (Sigma-Aldrich, St. Louis, MO) analysis via an enzymatic colorimetric assay adapted to a microtiter plate. Insulin analysis via chemiluminescent immunoassay (Siemens ADVIA Centaur, Deerfield, IL) was done by the UC Davis Medical Center’s clinical laboratory using a 1 ml sample from a SST tube. All samples were stored in a freezer at −30°C prior to analysis.

### Calculations and statistical analysis

Energy derived from total CHO and fat oxidation was calculated using the following equations, based on gas exchange measures of non-protein RER:

(1) $$\%\text{Energy from CHO}=\left[\left(\text{RER }–0.707\right)/0.293\right]\times \;100$$

(2) $$\%\text{Energy from Fat}=100–\left[\left(\text{RER }–0.707\right)/0.293\right]\times \;100$$

Data are presented as means ± standard deviation (SD). We employed a within-subject two-way analysis of variance (ANOVA) for repeated measures with a Fisher’s PLSD post hoc analysis to determine significant differences (StatView software, Version 5.0.1, SAS Institute Inc., Cary, NC). Significance was set at *p* ≤ 0.05.

## Results

### Subjects

Participant physical and training characteristics are presented in Table 1. The amount of calories consumed and macronutrient proportions from 3 day diet records were 2519 ± 405 kcal, 51 ± 7% CHO, 28 ± 6% fat, 16 ± 3% protein and 5 ± 4% alcohol. The 24-hr diet recalls prior to each trial showed 2368 ± 730 kcal, 56 ± 5% CHO, 27 ± 5% fat, 16 ± 2% protein and 1 ± 2% alcohol. The 24-hr diets were the same for all treatments.

**Table 1:** Subject physical characteristics

**Variable**	
Age, yr	29.3 ± 7.8
Height, cm	175.5 ± 3.9
Weight, kg	72.4 ± 11.1
Body fat, %	9.2 ± 4.4
Fat-free mass, kg	65.4 ± 7.3
Fat mass, kg	7.0 ± 4.8
VO_2_max	
1 min^-1^	4.2 ± 0.4
ml kg^-1^ min^-1^	58.2 ± 4.8
Training hours per week	8.0 ± 2.2
Running km per week	76.0 ± 13.5
Speed at max, km h^-1^	17.2 ± 1.6

Values are means ± SD for 11 men.

VO_2_, oxygen consumption.

### Physiological responses

**Figure 1:**
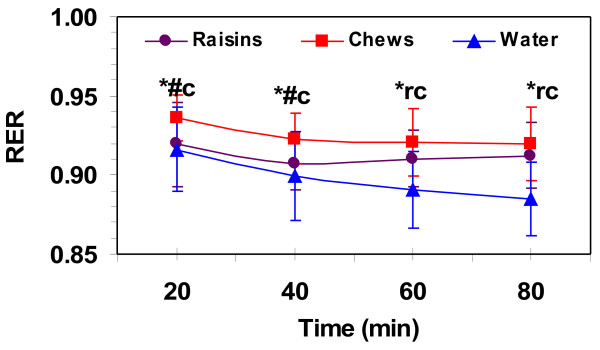
**Respiratory exchange ratio (RER) during 80-min of running at 75% VO**_**2**_**max.** Values are means ± SD for 11 men. *, significantly different from water and #, significantly different from raisin for (c) chews at 20 and 40-min and for (r) raisins and (c) chews at 60 and 80-min (*p* ≤ 0.05).

The treadmill speed averaged 13.2 ± 1 km·h^-1^ for all trials. VO_2_ did not change with time during the 80-min sub-maximal exercise bouts and averaged 44.3 ± 2.9, 44.2 ± 3.1, 43.7 ± 3.3 ml·kg^-1^ ·min^-1^ for raisin, chews and water respectively, with no difference between treatments. The percent of VO_2_max during the sub-maximal 80-min exercise bouts were 76.6 ± 4.4, 76.6 ± 4.4, 75.3 ± 5.1% for raisin, chews and water respectively, with no difference between treatments. Heart rate remained the same after 20-min of exercise for the entire 80-min sub-maximal exercise bout with the chews treatment, increased at 60- and 80-min for the water only trial and increased only at 80-min with the raisin treatment (Table 2). Average HR over the 80-min sub-maximal exercise bout was 158.8 ± 12.9, 160.1 ± 12.5, 157.4 ± 12.1 bpm for raisin, chews and water respectively, with no difference between treatments. RPE increased with exercise duration for all treatments (Table 2). However, there were no differences at any time point between treatments. RPE was rated as “hard” and averaged 4.8 ± 1.5, 4.9 ± 1.5, 5.2 ± 1.4 (0–10 scale) over the 80-min sub-maximal exercise bout for raisin, chews and water respectively. RER (Figure 1) decreased from 20 to 40-min for all treatments and then did not change for the rest of the 80-min sub-maximal exercise bout for any of the treatments. RER was significantly higher with the chews treatment than both water and raisins at 20-, 40- and 60-min of the 80-min sub-maximal exercise bout and both the chews and raisins were higher than water at 60- and 80-min of sub-maximal exercise. The % of energy from CHO decreased during the 80-min sub-maximal exercise bout with the water treatment, but remained stable after 40-min with the raisin and chews treatments (Table 2). The chews treatment had a higher % of energy from CHO and lower % energy from fat during the first 60–min of the 80-min of sub-maximal exercise than both water and raisins. Both raisins and chews had higher % of energy from CHO and lower % energy from fat at 60–min and 80-min of sub-maximal exercise than water. Body weight change from pre to post exercise did not differ between treatments and was −1.0 ± 0.4, -1.1 ± 0.3, -1.1 ± 0.4 kg for raisin, chews and water respectively.

**Table 2:** **Physiological responses to 80-min of Exercise at 75% VO**_**2**_**max**

**Variable**	**Raisins**		**Chews**		**Water**	
Heart Rate, beats min^-1^						
20 min	155.3 ± 14.4		158.0 ± 12.5		153.9 ± 14.9	
40 min	159.0 ± 12.0		160.5 ± 12.6		156.3 ± 12.6	
60 min	159.7 ± 12.8		160.6 ± 12.7		158.6 ± 11.8	†
80 min	161.2 ± 12.3	†	161.3 ± 12.1		160.7 ± 9.0	†
Exercise mean	158.8 ± 12.9		160.1 ± 12.5		157.4 ± 12.1	
RPE (0–10 scale)						
20 min	4.1 ± 1.8		4.0 ± 1.1		4.5 ± 1.5	
40 min	4.5 ± 1.5		4.8 ± 1.5		5.0 ± 1.3	
60 min	5.0 ± 1.4	†	5.1 ± 1.6	†	5.4 ± 1.3	†
80 min	5.5 ± 1.4	†‡	5.7 ± 1.7	†‡	5.9 ± 1.5	†‡
Exercise mean	4.8 ± 1.5		4.9 ± 1.5		5.2 ± 1.4	
% energy from CHO						
20 min	72.5 ± 9.1		78.2 ± 4.9	*#	71.3 ± 9.1	
40 min	68.1 ± 5.5	†	73.7 ± 5.4	†*#	65.6 ± 9.6	†
60 min	69.4 ± 6.2	*	72.9 ± 7.2	†*#	62.7 ± 8.2	†
80 min	70.1 ± 7.0	*	72.6 ± 8.0	†*	60.7 ± 7.8	†‡
Exercise mean	70.0 ± 7.0	*	74.4 ± 6.4	*#	65.1 ± 8.7	
% energy from Fat						
20 min	27.5 ± 9.1		21.8 ± 4.9	*#	28.7 ± 9.1	
40 min	31.9 ± 5.5		26.3 ± 5.4	*#	34.4 ± 9.6	
60 min	30.6 ± 6.2	*	27.1 ± 7.2	*#	37.3 ± 8.2	†
80 min	29.9 ± 7.0	*	27.4 ± 8.0	*#	39.3 ± 7.8	†
Exercise mean	30.0 ± 7.0	*	25.6 ± 6.4	*#	34.9 ± 8.7	

Values are means ± SD for 11 men.

*, significantly different from water; #, significantly different from raisins;

†, significantly different from 20 min; ‡, significantly different from 40 min

RPE, rate of perceived exertion; CHO, carbohydrate.

### Blood parameters

Hematocrit was not different between treatments before exercise (44 ± 3, 44 ± 3, 44 ± 2%, for raisin, chews and water respectively). Hematocrit increased from pre-exercise values for all treatments during the first 20-min, but remained the same for the rest of the sub-maximal exercise bout. Thus, we just report the average for the entire 80-min sub-maximal exercise bout. Hematocrit averaged 47 ± 3, 47 ± 3, 47 ± 3% for raisin, chews and water respectively, with no difference between treatments.

**Figure 2:**
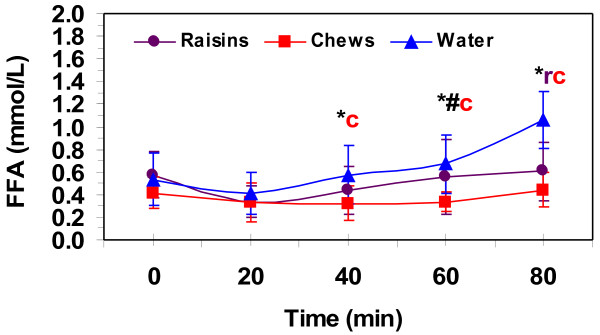
**Serum free fatty acid (FFA) levels pre-exercise and during 80-min of running at 75% VO**_**2**_**max.** Values are means ± SD for 11 men. *, significantly different from water and #, significantly different from raisin for (c) chews at 40 and 60-min and for (r) raisins and (c) chews at 80-min (*p* ≤ 0.05).

Metabolic responses averaged over the 80-min of exercise at 75%VO_2_max are presented in Table 3. Blood glucose was similar pre-exercise between treatments and only increased from rest at 40-min of the 80-min sub-maximal exercise bout in the chews treatment and at 80-min for the raisin treatment. Blood lactate was similar pre-exercise for all treatments and did not increase significantly above rest for the 80-min sub-maximal exercise bout for any treatment. Serum free fatty acid (FFA) concentrations (Figure 2) remained at pre-exercise levels for the entire 80-min sub-maximal exercise bout for the chews treatment, but increased significantly at 80-min compared to all time points for the water only treatment. The 20-min FFA was significantly lower than 60- and 80-min and the 40-min FFA was lower than the 60-min value for the raisin treatment. FFA was significantly higher with the water treatment compared to chews at 40-and 60-min of the 80-min sub-maximal exercise bout. Raisin was higher than chews at 60-min of sub-maximal exercise. Water had higher FFA than both raisin and chews at 80-min of sub-maximal exercise. Serum glycerol concentrations (Table 3) were not different at rest between treatments (0.09 ± 0.06, 0.11 ± 0.06, 0.12 ± 0.07 mmol·L^-1^ for raisin, chews and water respectively). Values increased for all treatments during exercise, but there were no differences between treatments.

**Table 3:** **Metabolic responses to 80-min of Exercise at 75% VO**_**2**_**max**

**Variable**	**Raisins**		**Chews**		**Water**
Glucose, mmol L^-1^					
Rest	5.3 ± 0.4		5.3 ± 0.4		5.3 ± 0.5
20 min	5.2 ± 0.9		5.7 ± 1.2		5.6 ± 0.6
40 min	5.8 ± 0.6		6.2 ± 0.7	$	5.7 ± 0.6
60 min	5.8 ± 0.7		5.8 ± 0.5		5.2 ± 0.6
80 min	5.9 ± 0.8	†	5.8 ± 0.6		5.2 ± 0.6
Exercise mean	5.6 ± 0.7		5.8 ± 0.7	*	5.5 ± 0.6
Lactate, mmol L^-1^					
Rest	1.1 ± 0.2		1.3 ± 0.4		1.2 ± 0.3
20 min	2.3 ± 0.6		2.6 ± 1.0		2.3 ± 0.9
40 min	2.1 ± 0.7		2.4 ± 1.2		2.3 ± 0.6
60 min	2.1 ± 0.5		2.2 ± 0.8		2.0 ± 0.5
80 min	2.1 ± 0.5		2.0 ± 0.6		2.0 ± 0.4
Exercise mean	1.9 ± 0.5		2.1 ± 0.8		2.0 ± 0.5
Glycerol, mmol L^-1^					
Rest	0.09 ± 0.06		0.11 ± 0.06		0.12 ± 0.07
20 min	0.11 ± 0.05		0.15 ± 0.08		0.14 ± 0.05
40 min	0.12 ± 0.06		0.14 ± 0.08		0.13 ± 0.06
60 min	0.13 ± 0.06		0.14 ± 0.07		0.14 ± 0.05
80 min	0.14 ± 0.05		0.14 ± 0.07		0.15 ± 0.06
Exercise mean	0.12 ± 0.05		0.14 ± 0.07		0.14 ± 0.06

Values are means ± SD for 11 men.

*, significantly different from water;

$, significantly different from rest; †, from 20 min.

**Figure 3:**
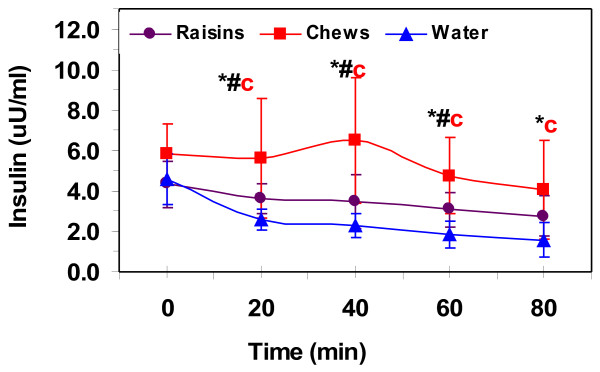
**Serum insulin levels pre-exercise and during 80-min of running at 75% VO**_**2**_**max.** Values are means ± SD for 11 men. *, significantly different from water and #, significantly different from raisin for (c) chews (*p* ≤ 0.05).

**Figure 4:**
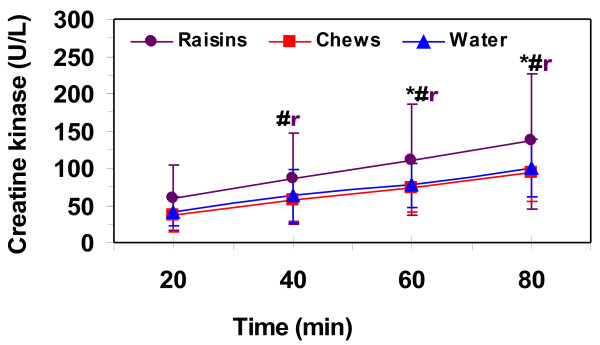
**Plasma creatine kinase (CK) levels, corrected for baseline, during 80-min of running at 75% VO2max.** Values are means ± SD for 11 men. #, raisin significantly different from water and chews (*p* ≤ 0.05).

Serum insulin values were similar pre-exercise at 4.3 ± 1.2, 5.8 ± 1.5, 4.6 ± 1.3 uU·ml^-1^ for raisin, chews and water respectively. Serum insulin during exercise (Figure 3) did not change significantly with the raisins, and was only higher at 40-min compared to 60- and 80-min of sub-maximal exercise for the chews. Insulin decreased with exercise compared to rest with water for all time points, but remained the same after 20-min. Insulin values were higher for the chews compared to water for all exercise time points and higher than raisins for the first 60-min of exercise. Pre-exercise plasma total CK levels were significantly higher with raisins than both water and chews at 328 ± 258, 210 ± 161, 219 ± 134 U·L^-1^ for raisin, chews and water respectively. These values were higher than the normal range for CK (38–174 U·L^-1^) and may reflect the high volume training protocols of our runners. Half of our subjects had pre-exercise CK levels above 174 U·L^-1^ for all treatments. Plasma CK, corrected from baseline values, increased during exercise for all trials and was higher after 40-min of exercise during the raisin trial compared to chews and after 60-min with water (Figure 4).

### Performance time trial

**Figure 5:**
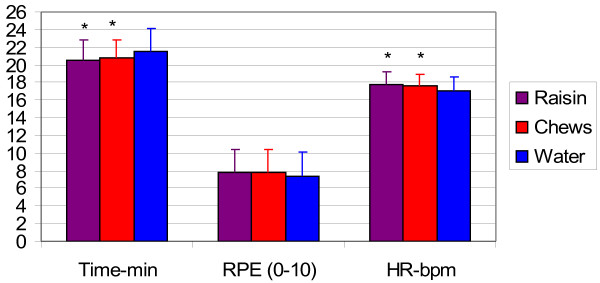
**Time of completion and average rate of perceived exertion (RPE) and heart rate (HR) (value/10) during the 5-km time trial.** Values are means ± SD for 11 men. *, significantly different from water (*p* ≤ 0.05).

Data from the 5-km TT is presented in Figure 5. Running time was lower by 1-min for both CHO treatments compared to water only (20.6 ± 2.6, 20.7 ± 2.5, 21.6 ± 2.7 min for raisin, chews and water respectively). While RPE was not different, HR was higher for both CHO treatments compared to water only during the 5-km TT.

### Questionnaires

**Figure 6:**
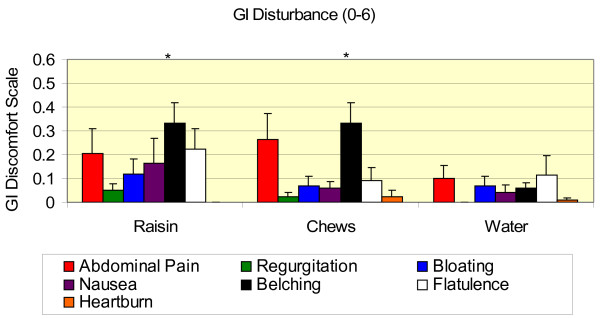
**Gastrointestinal disturbance by category over the entire exercise bout on a scale from 0–6 with 1 being mild and 6 being unbearable.** Values are means ± SD for 11 men. *, significantly different from water (*p* ≤ 0.05).

There were no differences due to treatment in the whole body soreness and fatigue questionnaires (Table 4), but all values increased over pre-exercise and remained higher 5-hr post-exercise. GI disturbance was very low for all categories (Figure 6). Values were averaged over the entire exercise trial including both sub-maximal exercise and the time trial. GI disturbance was in the mild range for all treatments. Belching was higher with both CHO treatments compared to water only.

**Table 4:** Data from Questionnaires

**Variable**	**Pre-Exercise**	**Post-Exercise**		**2-Hr Post**		**5-Hr Post**	
**Whole Body Muscle Soreness (out of 100 mm)**							
Water	15.4 ± 3.7	31.8 ± 5.2	+	34.5 ± 4.1	+	29.8 ± 3.7	+
Raisin	16.5 ± 4.2	35.3 ± 5.5	+	35.4 ± 5.2	+	34.0 ± 5.2	+
Chews	15.2 ± 3.8	37.4 ± 4.6	+	40.6 ± 4.9	+	40.6 ± 5.6	+
**Whole Body Fatigue (out of 100 mm)**							
Water	19.6 ± 4.8	50.4 ± 6.9	+	43.1 ± 4.2	+	42.9 ± 6.2	+
Raisin	23.7 ± 5.0	47.0 ± 6.2	+	43.2 ± 5.1	+	42.4 ± 3.9	+
Chews	21.4 ± 4.6	49.0 ± 6.9	+	43.6 ± 6.4	+	39.6 ± 7.1	+

Values are means ± SD for 11 men.

+, significantly different from pre-exercise.

## Discussion

Our results indicate that ingestion of a natural food product, raisins, had similar performance effects as a commercial sports product in chews and both products improved running time trial performance over water only. Raisins and chews maintained a higher % of non-protein macronutrient oxidation derived from CHO over the 80-min running bout at 75% VO_2_max than water only. The commercial product did cause slightly higher insulin levels and CHO oxidation rates during exercise than raisins. Raisins had a greater increase in creatine kinase during exercise than both chews and water only. Our data suggests that consuming a natural, relatively fiber-rich CHO source (raisins) had similar GI effects as a commercial product.

All treatments maintained blood glucose levels at pre-exercise values during the 80-min sub-maximal trials. However, the glucose levels during exercise were higher with the commercial product compared to water only. Similar glucose responses between carbohydrate forms is in agreement with a study examining the metabolic effects of raisins (glycemic index (GcI) = 62) versus sport gels (GcI = 88) in cyclists [10]. Even though at rest, high-GcI foods result in an elevation of plasma insulin concentrations compared to low-GcI foods, this has typically not been observed during exercise. Increased catecholamine levels typically suppress insulin release, even when CHO is consumed during exercise [18]. In our study, serum insulin levels were mostly unchanged during the exercise bout for the carbohydrate treatments and decreased during exercise in the water only trial. Insulin levels were higher for the commercial product during the first 60-min of exercise compared to both raisins and water only. This is in contrast to the study by Kern et al. where insulin levels were similar between raisins and sports gel after 45-min of cycling at 70% VO_2_max [10]. The feeding protocol was different in the Kern et al. study compared to ours in that the products were fed 45-min prior to exercise (ours ~10-min prior) and not given during exercise (we supplemented every 20-min of exercise). A slightly lower GcI (GcI = 62) with the raisins compared to chews (GcI = 88) may have contributed to the lower insulin response with raisins in our study.

Both CHO treatments produced higher RER values after 60-min of exercise, and thus greater energy contributions from CHO and less from fat compared to water only. Interestingly, the raisin treatment induced a lower energy contribution from CHO and greater from fat compared to the chews treatment. The slightly lower GcI may have decreased CHO absorption at the intestine and caused a slightly lower CHO oxidation rate with the raisins. The lower energy contribution from fat and higher from CHO with the chew treatment could have resulted from a type I statistical error, considering the small, non significant RER differences between raisins and chews during the last 20-min of exercise. Other studies support that relatively low-GcI foods do not have a different metabolic effect during exercise compared to high-GcI foods, especially when subjects receive carbohydrate supplements during exercise [10,18].

Preventing GI distress is important for competitive endurance performance. In our study, there was remarkably little to no adverse GI effects with all treatments. Studies have found an increase in GI symptoms experienced during running, which has been attributed to the mechanical jarring involved in running and the decreased blood flow to the GI tract during exercise [15,19]. GI blood shunting is dependent on exercise intensity, which can affect passive and active CHO absorption and delivery to the systemic circulation [20] and GI discomfort experienced during exercise. It has been found that at VO_2_max, both active and passive intestinal glucose absorption is significantly reduced compared to 30% and 50% VO_2_max [20]. Our subjects completed the 80-min running bout at ~75% VO_2_max, which may have reduced blood flow to the GI tract. However, the lower CHO consumption rate (~0.7 g·min^-1^) may have reduced the risk of developing GI discomfort. The recommended CHO consumption rate during exercise is 0.5-1.0 g·min^-1^[21]. Therefore, we speculate that the exercise intensity and amount of CHO consumed allowed for adequate GI blood supply to support high oxidation efficiency and a smaller % of the ingested CHO remained in the GI tract [1].

It was hypothesized that the increased fiber content in raisins, combined with the mechanical jarring involved with running, would result in greater GI discomfort. The dietary fiber in raisins could have had an osmotic effect in the intestinal lumen resulting in abdominal pain and diarrhea [14]. Our subjects consumed ~7 g·hr^-1^ fiber during the raisin treatment and had no severe GI disturbances compared to the chews and water treatments. A slight increase in belching was experienced for both the raisins and chews treatment yet, exercise performance was better in these trials than water only. There seems be a direct relationship between exercise duration and GI distress [15,22], especially in ultramarathon distances whereby GI distress can severely limit performance [22]. It is possible that if individuals continue to consume fiber-rich CHO sources, such as raisins, during endurance events >2-hr, the combined increase in exercise duration and fiber content in the GI tract could increase the severity of GI symptoms experienced. Further study with longer distances and in actual race conditions is warranted. Another factor that can contribute to GI discomfort is the hydration status of an individual. Subjects have reported GI complaints (37.5%) while exercising in a dehydrated state (4% BW loss) [23]. Hydration status in our subjects was sufficient in all treatments (hematocrit = ~47% and BW loss = ~1.5%), which could explain the few GI complaints.

The raisin treatment elicited higher plasma CK concentrations, corrected for baseline measures, during the 80-min run. We are unsure as to the causes of the higher CK values with the raisins, but only half of the subjects had higher responses with the raisin treatment compared to water or chews. The large standard deviations in the measurement of plasma CK levels could have played a role as could higher baseline levels before treatment consumption. The subjective scoring of muscle soreness and fatigue were similar between all treatments as was time trial performance and hydration status. Thus, the CK response to exercise appeared to be dissociated from other indices of muscle damage (e.g. muscle soreness and performance impairment) [24]. It is uncertain as to what factors resulted in the higher plasma CK concentrations with raisin ingestion and further research on the potential detrimental effects of raisin ingestion with exercise durations greater than 2-hours is needed.

This study is limited in that we conducted this experiment in the laboratory instead of an actual running competition and the treatments were given to subjects while standing on the treadmill instead of while running. Since the majority of runners in competition consume CHO while still running, it is uncertain whether solid CHO can negatively affect performance times solely by the act of consumption. Previous field studies have found that semi-solid CHO intake increased running time compared to liquid CHO intake [25]. There is the possibility that chewing solid CHO sources (e.g. chews and raisins) can disrupt an individual’s breathing pattern and in combination with running could negatively affect performance.

In conclusion, our study provides evidence that solid CHO consumption during a ~100-min run allows for maintenance of blood glucose levels and improved performance compared to water only. Our data suggests that consuming a natural CHO source (raisins) within the ACSM/ADA/DC recommendations [21] is well tolerated and maintains blood glucose levels and running performance similar to a commercial CHO product (sport chews).

## Competing interests

The authors declare that they have no competing interests.

## Authors’ contributions

BT participated in the design of the study, recruitment of subjects, data collection, data analysis and drafted the manuscript. SC assisted in the design of the study, recruitment of subjects, data collection and data analysis. KH assisted in the recruitment of subjects, data collection and data analysis. LA participated in the design of the study and manuscript preparation. BD participated in the design of the study and manuscript preparation. GC participated in the design of the study, data collection, data analysis, statistical analysis and helped draft the manuscript. All authors read and approved the final manuscript.
